# Effects of Pollution on Pregnancy and Infants

**DOI:** 10.7759/cureus.33906

**Published:** 2023-01-18

**Authors:** Prerna Rani, Archana Dhok

**Affiliations:** 1 Medicine, Jawaharlal Nehru Medical College, Datta Meghe Institute of Higher Education & Research, Wardha, IND; 2 Biochemistry, Jawaharlal Nehru Medical College, Datta Meghe Institute of Higher Education & Research, Wardha, IND

**Keywords:** pollutants, myotoxins, infants, pollution, pregnancy

## Abstract

The fetus is particularly susceptible to environmental contaminants as it is developing at the time of pregnancy and is, therefore, more susceptible to their effects. Pregnancy loss, which includes stillbirth and spontaneous abortion (miscarriage), preterm labor and delivery, and neonatal death, is the worst pregnancy outcome. Stunting and its related health and developmental effects are particularly common in populations living in underdeveloped countries or those exposed to high levels of particle pollution. Several environmental toxins can affect an embryo, fetus, or infant as they are developing. This study explores the following questions: What part do pesticides, heavy metals, dioxin derivatives, and polychlorinated diphenyl compounds play as macroenvironmental pollutants in mutagenesis and teratogenesis? What effects do substances that exposed persons have considerable control over, such as alcohol, narcotics, and tobacco smoke, have on the microenvironment? What consequences should practitioners be aware of these toxins in terms of ethics and the law? This study seeks to assess pertinent primary scientific studies on how pollution affects the health of the fetus and newborn during pregnancy.

## Introduction and background

Future health may be impacted by environmental pollutant exposure during pregnancy and fertilization. Pregnancy and infancy are crucial periods of vulnerability. The metal buildup in the placenta can limit the fetus's growth, resulting in preterm birth, limited fetal growth, increased gestational diabetes, shorter telomere length, higher uterine vascular resistance, and reduced chromosomal stability. Placental vascularization is one of the many well-known harmful effects of air pollution on pregnancy [1]. In recent years, the amount of research on how pollution affects human health has grown dramatically. Many studies have demonstrated that exposure to pollutants during pregnancy, a critical period for mother-fetus development, may have a major, lasting effect on an unfavorable pregnancy [2]. One of the main causes of sickness and mortality that may be both restricted and managed is exposure to air pollution, which can occur in both open and enclosed places. There are several disagreements and discrepancies among the studies that have examined the impacts of prenatal pollution exposure, despite the fact that the body of evidence is expanding [3]. Due to the potential exposure of the developing fetus, environmental toxins have a significant and particularly negative effect on the human population during pregnancy. According to the Developmental Origins of Health and Disease hypothesis, a fetus's susceptibility to environmental elements that may have an impact on how diseases manifest themselves later in life can have a significant impact on those elements [4].

The fetus is particularly susceptible to environmental contaminants as it is developing at the time of pregnancy and is, therefore, more susceptible to their effects. The worst pregnancy outcomes include stillbirth and spontaneous abortion (miscarriage), which are serious morbidities for the mother. This review focuses on how air pollution exposure affects spontaneous abortion and stillbirth during pregnancy [5]. By calculating the direct and indirect effects on results, causal mediation analysis assists in finding the causal processes or effect pathways (i.e., effect decomposition). Metabolites and metabolic pathways in the mother are affected by air pollution, which suggests that they may function as mediators in the pathways that produce unfavorable pregnancy and birth outcomes. There is some evidence that macro-toxins can have bad effects on a pregnancy. Lower birth weight, neonatal jaundice, fetal death, maternal anemia, and other undesirable outcomes have been identified as possible effects. Metabolomic markers have been used to elucidate the molecular pathways linking arsenic exposure to birth weight as well as the connection between air pollution and respiratory function [6]. The *Developmental Origins of Health and Disease* idea contends that the early experiences of a fetus have a profound impact on its programming. Epigenetic changes and nitrosative stress in the placenta are linked to prenatal air pollution exposure. Only a few physiological functions, such as DNA repair, circadian rhythm, and energy metabolism, were adversely affected by CpG sites. The expression levels of the miRNAs miR-20a, miR-21, miR-146a, and miR-222 varied according to the level of air pollution to which they were exposed. Early life aging indicators like telomere length and mitochondrial DNA content are connected to prenatal air pollution exposure [7].

## Review

Methodology

**Figure 1:**
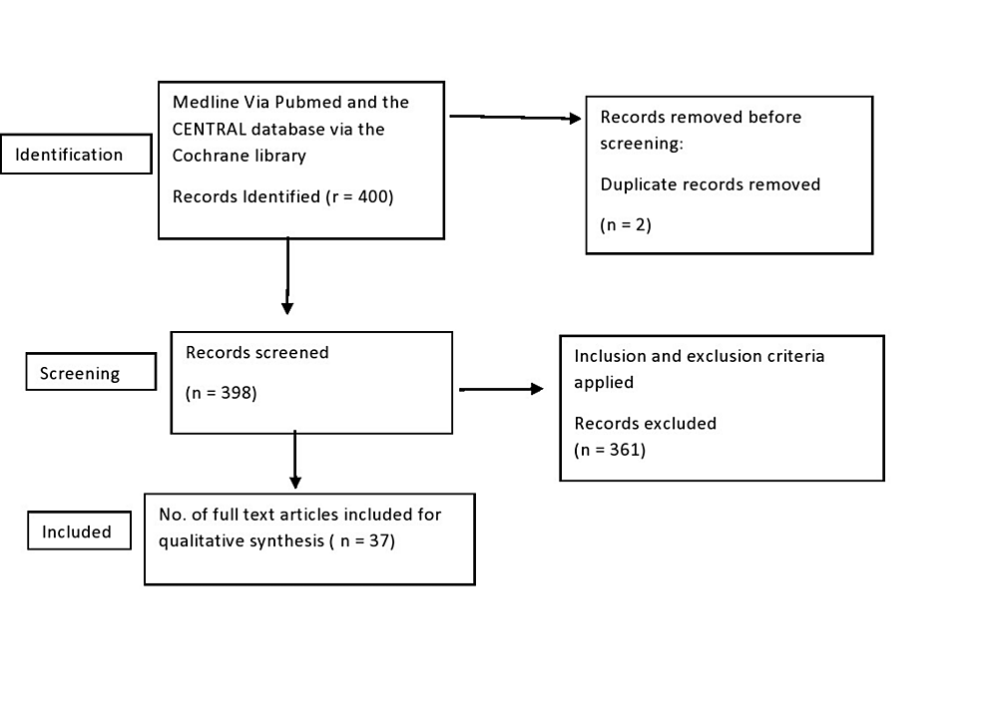
Identification of search strategies for qualitative review synthesis databases and registers on the effects of pollution on pregnancy and infants. Figure credits: Prerna Rani.

We used the Cochrane Library and PubMed to search the Central database and Medline. The following search method was specifically designed for each database: Effects of pollution on pregnancy and infants. To find new studies, we also looked through the references list of potentially relevant papers. Studies returned from these computerized searches and pertinent sources listed in those studies' bibliographies were examined (Figure 1).

Effect of air pollution

Exposure to air pollution throughout the first 1,000 days of life (from conception to the second year of life) may be particularly important for children's long-term health. In addition to serving as indications of previous exposures, changes in biological markers, such as DNA methylation and telomere length, may contribute to the relationship between exposure to air pollution and illnesses associated with it [8]. Air pollution may indirectly harm lung development by causing low birth weight, early birth, or improper immune system development. The health implications of this exposure are especially important as air pollution during the prenatal period may interfere with organ development and organogenesis. Infant and child lung function impairment, an increase in respiratory symptoms, and the advent of childhood asthma have all been linked to prenatal exposure to air pollution [9]. When particulate matter (PM) 2.5 exposure was increased, similar small for gestational age (SGA) effects were observed throughout the whole pregnancy, including the second and third trimesters (PM). The respiratory health of a pregnant woman can be impacted by several variables, including air pollution. A 10 g/m^3^ increase in fine PM 2.5 exposure during pregnancy was significantly correlated with SGA (odds ratio [OR] = 1.08; 95% confidence interval [CI] 1.03-1.13). Additionally linked to postnatal exposure to household air pollution was a 19% greater incidence of postnatal stunting (95% CI 1.10-1.29). Our research demonstrates that exposure to indoor air pollution and ambient PM 2.5 increases the risk of health issues connected to stunting [10].

Effect of nicotine on the fetus

Pregnant women who smoke or use tobacco in other ways have a higher risk of obstetric issues and bad consequences for the unborn child counseling interventions are not as effective as incentive-based programs, which seem to considerably promote quitting smoking [11]. Childhood respiratory diseases are a significant contributor to morbidity and mortality, especially in low- and middle-income countries (LMICs). Long-term and short-term respiratory disorders are both known to be at increased risk due to ETS exposure. Seventy percent of youngsters (aged 14-20 years) over the world are exposed to ETS, while the true prevalence may be underreported. Infant lung development is impacted by maternal smoking and ETS exposure, and these issues are connected to teen upper and lower respiratory infections, wheezing, or asthma [12]. Both the likelihood that a woman will smoke while having a child and any potential risks that smoking may present to the fetus is significantly influenced by the genotype of the mother. Children's forced expiratory flows, passive respiratory compliance, hospitalizations for respiratory infections, and prevalence of asthma and wheezing are among the main effects of maternal smoking on children's lungs and health. By taking more vitamin C, some of the negative effects of maternal smoking on lung development can be mitigated. It is thought that nicotine, which is present in cigarette smoke, is what causes these adverse consequences. Because nicotine is the primary ingredient in e-cigarettes, using them while pregnant is likely to be just as bad for the fetus [13].

Effect of noise pollution on infants

Loud noise is pervasive and has become the norm as a result of growing urbanization and changing lifestyles. Environmental noise pollution is now understood to be a severe health risk, with growing negative impacts on fetuses, infants, kids, teenagers, and adults. Both indoors and outdoors are subject to this risk. People of all ages, even fetuses, are increasingly being found to have noise-induced hearing loss and other negative impacts. The long-term impairment brought on by noise pollution has been exacerbated by older motorized vehicles, machinery, increased traffic, congested residential areas, workplaces, and unrestrained commercial and industrial noise [14]. According to six cohorts, four case-control, and two cross-sectional studies, chronic noise exposure during pregnancy was not linked to low birth weight, preterm birth, congenital abnormalities, or perinatal or neonatal mortality. Chronic noise exposure was linked to higher levels of stress hormones, saliva and urine, and systolic blood pressure in babies, according to cross-sectional data from 15 research and two cohort studies [15]. According to the research, being exposed to occupational and, to a lesser extent, ambient noise may cause a modest rise in the prevalence of congenital malformations. No conclusions could be reached as there were few research that examined perinatal mortality and those who survived adopted various standards of success (in reducing noise pollution). However, a recent major cross-sectional investigation found a link between traffic noise and stillbirth. Several studies point to a potential link between congenital hearing loss, workplace noise exposure, and pregnancy [16].

Effect of environmental toxins on pregnancy

Endometriosis in female offspring may become more common if pregnant women are exposed to estrogenic drugs such as ethinyl estradiol and diethylstilbestrol as well as environmental contaminants such as bisphenol A, polychlorinated biphenyls, and 2,3,7,8-tetrachlorodibenzo-*p*-dioxin. But antiestrogenic properties of cigarette smoke might prevent female kids from getting endometriosis [17]. Pregnant women should refrain from eating fast food, drinking alcohol, or using tobacco products, all of which are known to be damaging to human health, as well as toxic natural compounds such as mycotoxins. Mycotoxins are organic toxins that can take on various chemical forms. In addition to directly consuming agricultural products, people can also be exposed to these poisons indirectly through items created by animals that were fed contaminated feed. The mutagenic, teratogenic, and carcinogenic properties of mycotoxins are harmful to human health. Some data suggests that mycotoxins can negatively impact a pregnancy. These potential unfavorable factors include lower birth weight, neonatal jaundice, fetal loss, fetal anomalies, preterm birth, maternal anemia, and hypertension [18]. When compared to the World Health Organization (WHO) standard growth curves, aggregate assessments of child growth in LMICs portray an alarmingly constant picture of serious development failure. Poor diets with little variety in food are a major contributor to slow development, but other significant environmental factors also place restrictions on proper nutrient intake and use. In this study, these elements are taken into account. It is possible to attribute a significant percentage of the rapid development degradation in later infancy to infections as well as more general, nonspecific impacts of living in an unclean environment, including consuming poisons like aflatoxin [19]. The decidua and blastocyst interact biochemically to determine the early pregnancy stage. A miscarriage or other unfavorable pregnancy outcomes could result from any modification to this chemical exchange. The most frequent pregnancy problem, sporadic miscarriage, may have various causes. There are, undoubtedly, additional contributing factors that could lead to an early loss, although genetic problems in the fetus are the most frequent cause of miscarriage [20]. The epigenetic effects of some environmental contaminants may have transgenerational health consequences. Examining the epidemiological and experimental evidence implicates some pollutants such as pesticides, flame retardants, perfluorinated compounds, and plasticizers as having detrimental impacts on reproductive health [21].

Effects of mercury exposure on pregnancy

Mercury (Hg) is a dangerous heavy metal element that can accumulate in plants and animals and change into methylmercury (MeHg). MeHg is a widely dispersed environmental contaminant that may impact populations of fish eaters by enhancing aquatic food chains. The major focus of MeHg is the central nervous system. Being more susceptible to MeHg than adults, embryos and young children can suffer long-lasting neurodevelopmental abnormalities from exposure to MeHg during prenatal nutrition [22]. Numerous longitudinal studies of the afflicted groups suggest that early exposure to MeHg can have long-lasting impacts that are likely caused by altered developmental processes. It is crucial to research neurobehavioral changes using ecologically realistic and physiologically relevant models given the sizeable number of newborns at a higher risk of learning impairments linked with in-utero MeHg exposure [23].

Effect of water pollution on pregnancy

There is proof that several widely used products include toxins that might harm reproduction in infancy, adolescence, and even adulthood. These substances might impair hormone synthesis and/or function negatively, which could affect future generations [24]. Studies have shown that consuming tetrachloroethylene (PCE)-contaminated water as a baby can have long-term neurological effects. Bipolar disorder, substance usage, and post-traumatic stress disorder were found to have the strongest correlations. The study's main benefits are a high incidence of exposure, a wide range of exposure levels, low confounding, and historical information on the impacted water systems. The historical element of the exposure evaluations was the primary source of difficulties [25].

Allergies caused by pollution

It's critical to recognize the risk factors for atopy as early as possible in life given the increased prevalence of allergy issues in kids. Since pregnancy, it may be feasible to anticipate the onset of atopic dermatitis and even food allergies. Children's atopic illness development and clinical symptoms are complicatedly influenced by a complex interplay between genetic and environmental factors, including allergen exposure, air pollution, and infections [26]. Asthma prevalence has dramatically increased since the early 1970s, and this increase can only be explained by changes in how people are exposed to environmental factors and changes in lifestyle. The probability of developing allergic asthma increases with allergen exposure due to the potential for increased allergic sensitization. There is insufficient data to show a causal link between allergen exposure and the development of the disease, except for indoor mold exposure, which is a risk factor for pediatric asthma [27]. Early life exposures have been discovered to have a substantial impact on the development of allergy diseases. Several prenatal and early life factors that have been linked to the emergence of pediatric allergies are listed in this study. Discussion topics include a delivery method, the gut microbiome, vitamin D, folate, lactation, pets, antibiotics, ambient cigarette smoke, and airborne traffic pollution. Overall, there are no factors that blatantly increase or decrease the incidence of allergic outcomes although many studies imply an influence of risk factors [28]. Regarding the link between bisphenol A (BPA) exposure and the emergence of childhood asthma, there is conflicting evidence. Our research looked at the epidemiological evidence linking perinatal or prenatal BPA exposure to a higher risk of developing asthma or wheezing in children [29].

Examples of chronic obstructive respiratory diseases that typically begin in childhood include asthma and chronic obstructive pulmonary disease. Early environmental exposures during pregnancy and youth, in addition to a genetic predisposition, have a lasting impact on respiratory health. These elements, which are present during a critical period of lung development, may alter the metabolism and morphology of the lungs and result in maladaptive responses to potentially harmful compounds, which affect an individual's longevity [30].

Early Lead Exposure and Child Neurodevelopment in the Presence of Other Neurotoxicants

While it is normal to be exposed to several nortriptyline (NT) drugs and environmental lead (Pb) at the same time, it may be difficult to foresee how these two substances would interact during infancy. We have focused much on Pb, and it also contains other pollutants such as metalloids, halogens, organohalogens, and other metals such as manganese (Mn), mercury (Hg), aluminum (Al), and cadmium (Cd). Co-occurring Pb-related exposures during pregnancy and lactation rely on environmental sources, metabolism, and the half-life of the specific NT pollutant, even if social stressors also have an impact on children's neurobehavioral outcomes [31].

Effect of Environmental Phthalate Exposure on Pregnancy Duration and Newborn Outcomes

The study's results highlight the need for public health actions to minimize phthalate exposure and offer more proof that exposure to phthalates may be associated with a smaller head circumference and a shorter gestational period [32].

Lung Development and the Physicochemical Environment

Changes in lung expansion cause mechanical stresses on lung tissue, which are a major determinant in fetal lung development. These forces have a considerable impact on the pace of cellular proliferation, the level of alveolar epithelial cell differentiation, and the three-dimensional tissue structure. Although the types of mechanical forces operating on the air-filled lung are known to be more different and more complex, they are thought to have a similar effect on lung growth after the air-filled lung has been removed [33].

Effects of Aromatic Hydrocarbon Pollution on the Endocrine and Metabolic Processes of the Human Placenta

**Figure 2:**
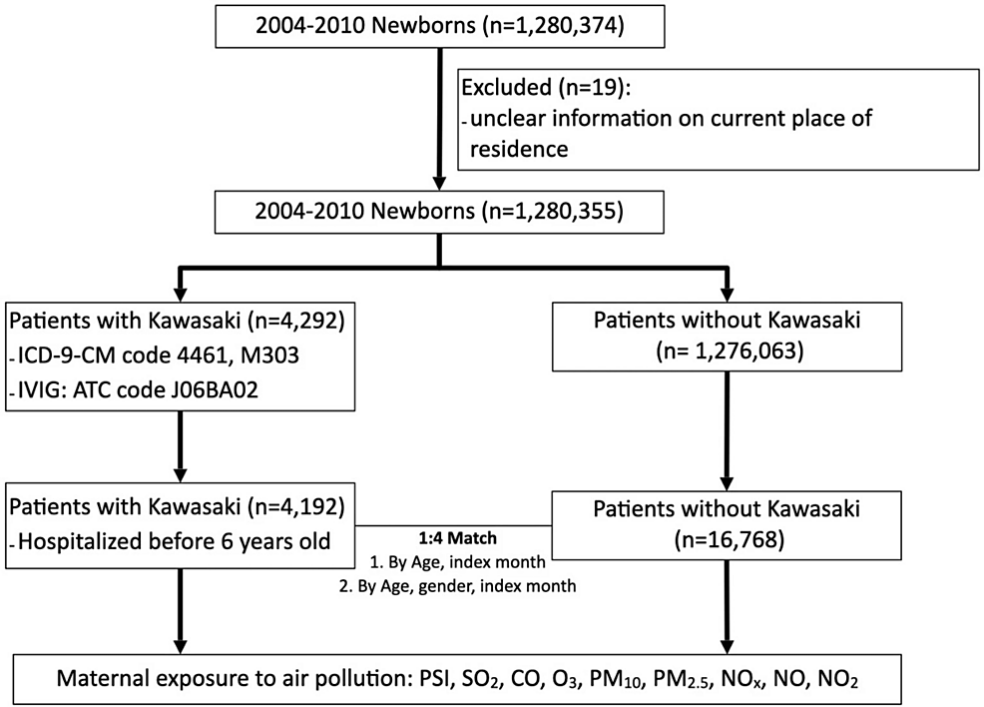
Prenatal and early exposure to air pollution and the incidence of Kawasaki disease. Source: [37]. ICD-9-CM, International Classification of Diseases, Ninth Revision, Clinical Modification; IVIG, intravenous immune globulin; ATC code, Anatomical Therapeutic Chemical code; PSI, Pollutant Standards Index; SO_2_, sulfur dioxide; CO, carbon monoxide; O_3_, ozone; PM, particulate matter; NO, nitric oxide; NO_2_, nitrogen dioxide

The patients who are affected by phenol and 1-hydroxypyrene concentration are having higher chances of voiding urine, which is controlled by the pons region. Placental phosphatase activity and placental glutathione transferase expression were statistically lower in pocked placentas. It has been shown that placentas from regions free of aromatic hydrocarbon contamination express higher levels of placental gonadotropin and estrogen receptor activity [34]. Poor prenatal development and oxidative stress are caused by environmental toxins and lifestyle choices. Currently, a major public health issue is developmental toxicity brought on by exposure to different environmental toxins. In addition to harmful lifestyle choices such as smoking, drinking, and using prescription medicines, human-made toxins like xenoestrogens, pesticides, and heavy metals have a detrimental effect on fetal development and increase the risk of sickness in offspring [35]. Environmental factors that affect how arsenic is methylated in individuals are arsenic exposure level, age, BMI, sex, smoking, genetic factors, ethnicity, pregnancy, and breastfeeding [28,29]. Despite having little daily intraindividual variation, subjects exposed to arsenic have considerable interindividual variability in their urine patterns of arsenic metabolites. Some of the differences in sensitivity to arsenic poisoning may be explained by the variable in arsenic methylation between individuals. A genetic influence on arsenic metabolism is indicated by the substantial interethnic heterogeneity and familial relationships in urine arsenic (Figure 2) [36].

## Conclusions

We should foster an atmosphere where each child may grow up healthy and exhibit the necessary skills to contribute to society if we want to preserve a prosperous and sustainable society. The following measures can be taken into consideration: It is necessary to locate noisy areas and implement remedies. Toys and residential, commercial, and industrial machinery should all be within a safe sound range. The use of loudspeakers and car horns should be restricted to emergencies only. Noise pollution at night must be kept away from residential areas since it can seriously harm long-term health. It is advised that pregnant women limit or prevent exposure to air pollution, especially during the early and late stages of pregnancy. Priority should be placed on policies that reduce maternal exposure and the consequences for the health of children. Minorities and people with low-socioeconomic status are recognized to be more exposed to PM 2.5 than other groups, although its levels are managed. To reduce maternal exposure and eventually safeguard the health of children, preventive interventions that guide neighborhood/regional planning and nutrition advice are required. Numerous actions have been taken to restrict exposure to dioxins, and this has led to a decrease in the amount of dioxin in breast milk. Nevertheless, additional steps must be taken to limit exposure. It is recommended to take legislative actions to lessen the release of PCBs, in particular, from garbage into the environment, to lessen developmental exposure and its effects, and individual preconception counseling is advised. It is advised to monitor both the chemical potential endocrine effects and their biomonitoring in meals and breast milk. Fast food, alcohol, and cigarette products, which are already to known be dangerous to human health, should be avoided by expectant mothers along with toxic natural items that include mycotoxins.
